# Hospital Administration

**Published:** 1899-09-09

**Authors:** 


### HOSPITAL ADMINISTRATION.

### THE LEEK COTTAGE HOSPITAL.

We are very sorry to find that certain difficulties have arisen in regard to the use of the Leek Cottage Hospital, and we are still more sorry to observe the angry tone in which the correspondence on the subject in the local press has been carried on. We do not pretend to go into the reasons which have led the committee to refuse to allow Dr. Hammersley to attend upon cases in this hospital, but certain points arise out of the discussion which seem to us to be fairly obvious and to be worthy of comment. The first is that if Dr. Hammersley is correct in saying that the resident staff of the hospital consists of "a matron, housemaid, and cook," and that the housemaid at the hospital, "a young woman engaged as a housemaid, and paid as a housemaid," has to be "present and assist at operations of the most delicate nature performed on male patients," the hospital organisation is evidently arranged on a basis such as, however permissible twenty years ago, or even now in distant colonies where no better arrangements can be made, is distinctly unfitted for England in the present day. Dr. Hammersley says, "In the hospital there are practically no appliances. There is not a single surgical instrument of any description. There is not the most simple apparatus for carrying out the often necessary examinations in medical cases. There is not a lamp for the examination of throat or eye, &c. There is not a prescription paper, nor even a temperature chart; and the record of a patient's illness, the temperature, pulse, &c., is never kept."

In answer to these charges, the hon. secretary says that other members of the medical staff say that the nursing is sufficient, that "as the medical officers attend to their own private patients in the hospital, it has always been understood that they prefer to bring their own instruments," and that "the committee do not deem it part of their duty either to provide surgical instruments or medicines, or in any way supervise either the medical or surgical treatment of the patients, who are simply housed and nursed in the hospital instead of in their own homes, the attendant medical officer being their own medical man, who provides his own instruments and medicines, and whose services they pay for independently of the hospital."

Now, of course, if the good people of Leek know all this, and if in that town the word "hospital" has a local and peculiar significance, which makes it suggest to the people of the district an institution such as is described above, it is perfectly open to the managers to run it on that basis. To most people, however, the term "hospital" conveys a very different meaning, and we think that in view of the explanation given by the hon. secretary there is a good deal to be said for the correctness of Dr. Hammersley's description when he speaks of it as "an anachronism."

Another thing, however, that is clear is that a committee must always be held responsible for the management of the institution which it controls, and that a patient entering such an institution must be taken to thereby submit himself to the properly constituted rules. It seems to us then, that if the committee were acting within their rights in removing Dr. Hammersley's name from the list of medical officers of the hospital, he was in the wrong in saying in his letter to the secretary, "I intend to visit my patient until the committee prevent me so doing by force." There are proper ways of deciding whether the committee has acted legally, and in no case should access to a hospital become a matter of using force or committing an assault.

Finally, it seems to us quite clear that when a patient went to the hospital and thus submitted himself to the committee and its rules, and when Dr. Burnett (another of the medical officers) was notified to attend this patient, as being surgeon for the week, he was perfectly justified in so doing. There can be no medical etiquette which would prevent the duly appointed medical officer to a hospital attending a patient admitted to it on the ground that the patient had been under the care of an outside practitioner. Whether Dr. Hammersley is now an outside practitioner, or is still a member of the staff, must be decided either by the committee or by legal process, but we think that the decision of the committee must be accepted until it is upset.

